# Supplementary material for: GRiNCH: simultaneous smoothing and detection of topological units of genome organization from sparse chromatin contact count matrices with matrix factorization
Source: Genome Biol. 2021 May 25;22:164. doi: 10.1186/s13059-021-02378-z (PMC8152090; doi:10.1186/s13059-021-02378-z)
Supplement: Supplementary file 1 — Additional file 1 Supplementary_figures.pdf. PDF file containing Figures S1-S18. [file 13059_2021_2378_MOESM1_ESM.pdf]

List of Supplementary Figures

S1 Selecting hyperparameters in GRiNCH. 2
S2 Distribution of TAD lengths for different methods across resolutions. 3
S3 Similarity of TADs across resolutions measured by mutual information 4
S4 Similarity of TADs from different TAD-calling methods 6
S5 Histone modification enrichment in TADs across different resolution. 7
S6 Similarity of topology and significant interactions among Hi-C data using different restriction enzymes and smoothed by GRiNCH. 8
S7 Similarity of GRiNCH TADs from mouse neural development time-course Hi-C data 9
S8 Interaction and regulatory profile near Arl6ip5 and Foxp1 during mouse neural development 10
S9 Visual comparison of TADs around Zfp608 during mouse neural development 11
S10 Visual comparison of TADs around Syap1 and Ap1s2 during mouse neural development . . 12
S11 Visual comparison of TADs around Arl6ip5 and Foxp1 during mouse neural development . . 13
S12 Similarity of GRiNCH TADs from pluripotency reprogramming time-course Hi-C data . . . 15
S13 Interaction and regulatory profile near Sox2 during mouse pluripotency reprogramming . . . 16
S14 Characterizing GRiNCH clusters of different size scales 17
S15 Enrichment of regulatory signals in GRiNCH clusters of different size scales 18
S16 GRiNCH TAD size distribution by regularization parameters 20
S17 Memory consumption and runtime trend of GRiNCH algorithm 21
S18 Comparison of NNDSVD initialization versus random initialization 22

Supplementary Figure 1

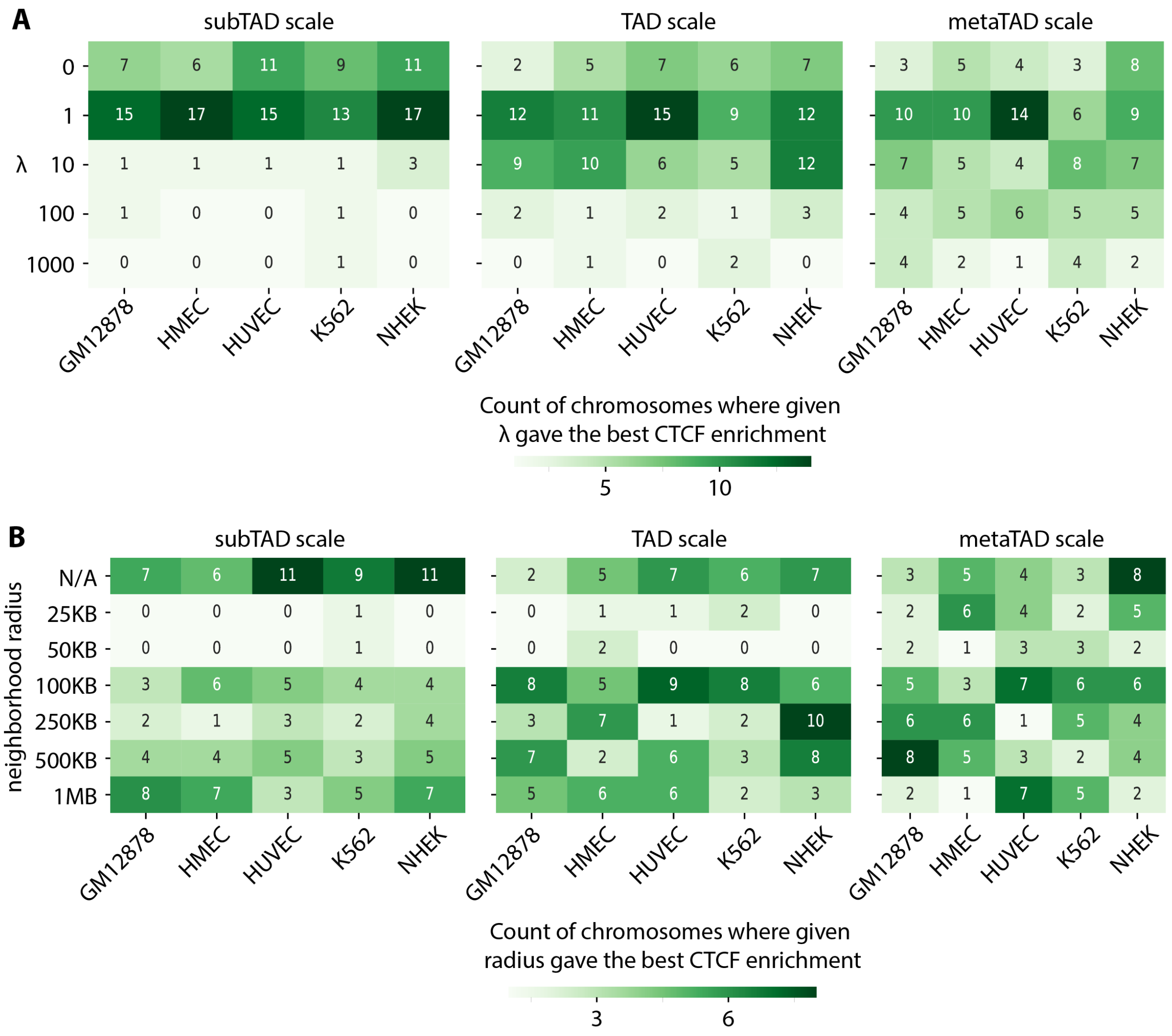

Figure S1: Selecting graph regularization parameters λ and neighborhood radius, r. λ controls the strength of regularization. r determines how many neighboring genomic regions will be used to influence a given region during the regularization process. **A**. Shown are the count of chromosomes in which the given λ value (row) gave the best CTCF enrichment within the given cell line (column), for different k settings denoted by subTAD, TAD, and metaTAD scale (see **Figure S14**, **Figure S15**). Within each cell line, for each chromosome, we ranked the tested parameter combinations of λ and r, and then counted the times a given λ yielded the best CTCF enrichment (regardless of r value). Due to ties in ranking, each column can add up to 23 or more. $\lambda = 0$ corresponds to vanilla NMF without regularization. **B**. Shown are the count of chromomes in which the given neighborhood radius value (row) gave the best CTCF enrichment in each cell line (column) at different k settings. As in **A**, we rank the parameter combinations of λ and r based on the CTCF enrichment, then count the number of times a particular value of r yielded the best fold enrichment. N/A corresponds to vanilla NMF without regularization.

Supplementary Figure 2

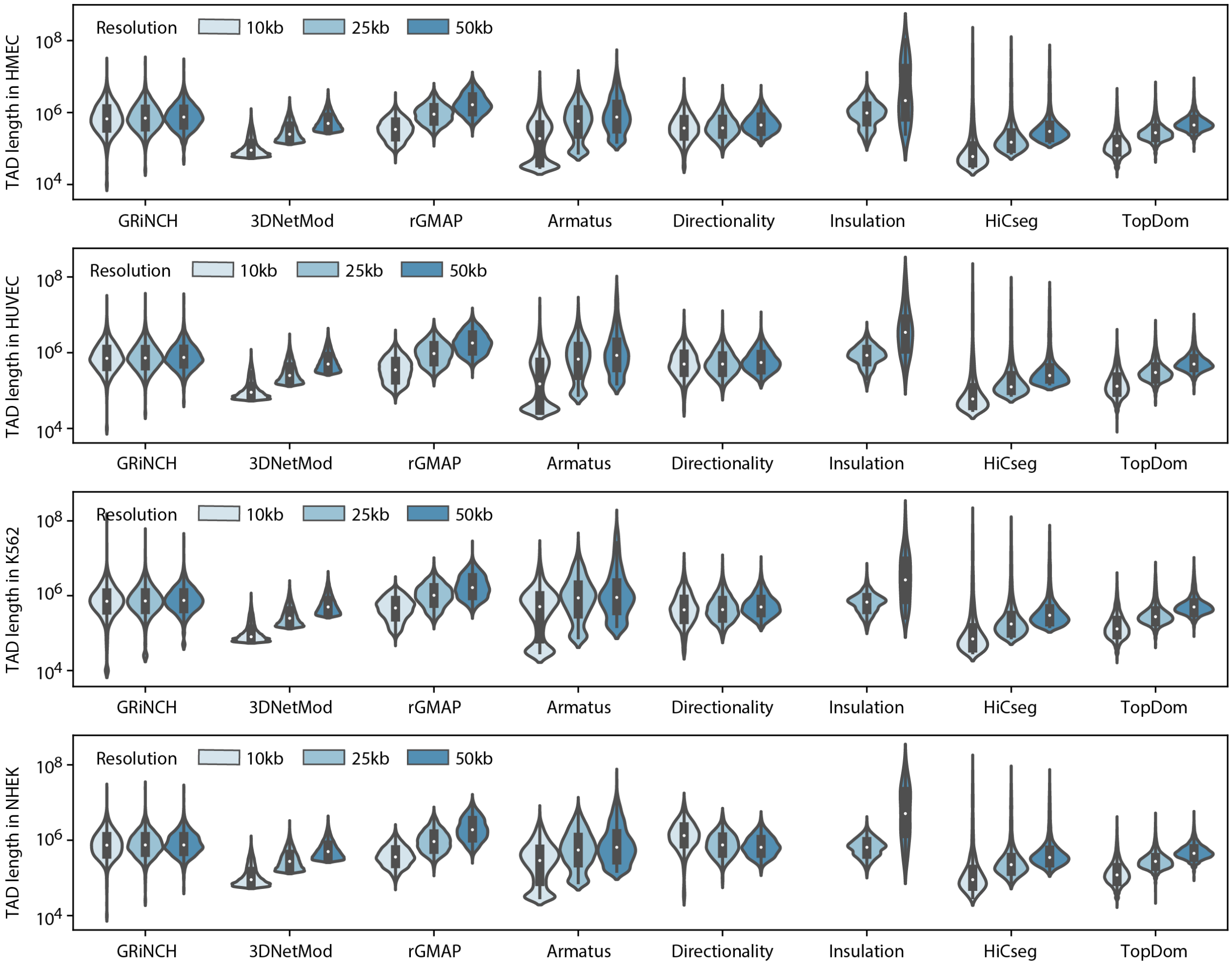

Figure S2: The size distribution of TADs identified by different methods from different resolutions of Hi-C data. Y-axis is in log10 scale of base pairs. The white dot in inside each violin represents the median; the black box inside each violin stretches from Q1 (25th percentile) to Q3 (75th percentile). Insulation method is missing TAD distributions from 10kb data because it did not return any TADs when using the same hyperparameters as in from 25kb and 50kb data.

Supplementary Figure 3

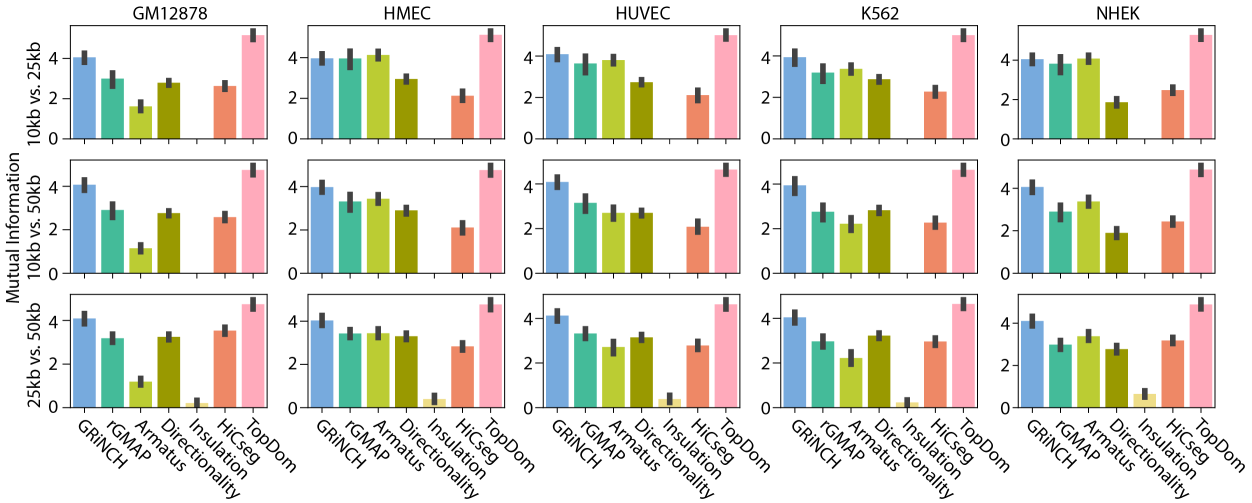

Figure S3: Similarity of TADs across resolutions for different methods, measured by mutual information. TADs were first converted to clusters, by assigning regions within the same TAD into a single cluster. To compare across resolutions, 10kb, 25kb, and 50kb bins were split into a size of lowest common denominator, i.e., 5kb. Then all 5kb bins were assigned to the same cluster as in the original lower-resolution bin (e.g. a 10kb bin assigned to cluster A would yield two 5kb bins assigned to cluster A). Finally, cluster assignments in these split, higher-resolution bins were compared for their similarity with Rand Index (Figure 3 in main text) and Mutual Information.

Supplementary Figure 4

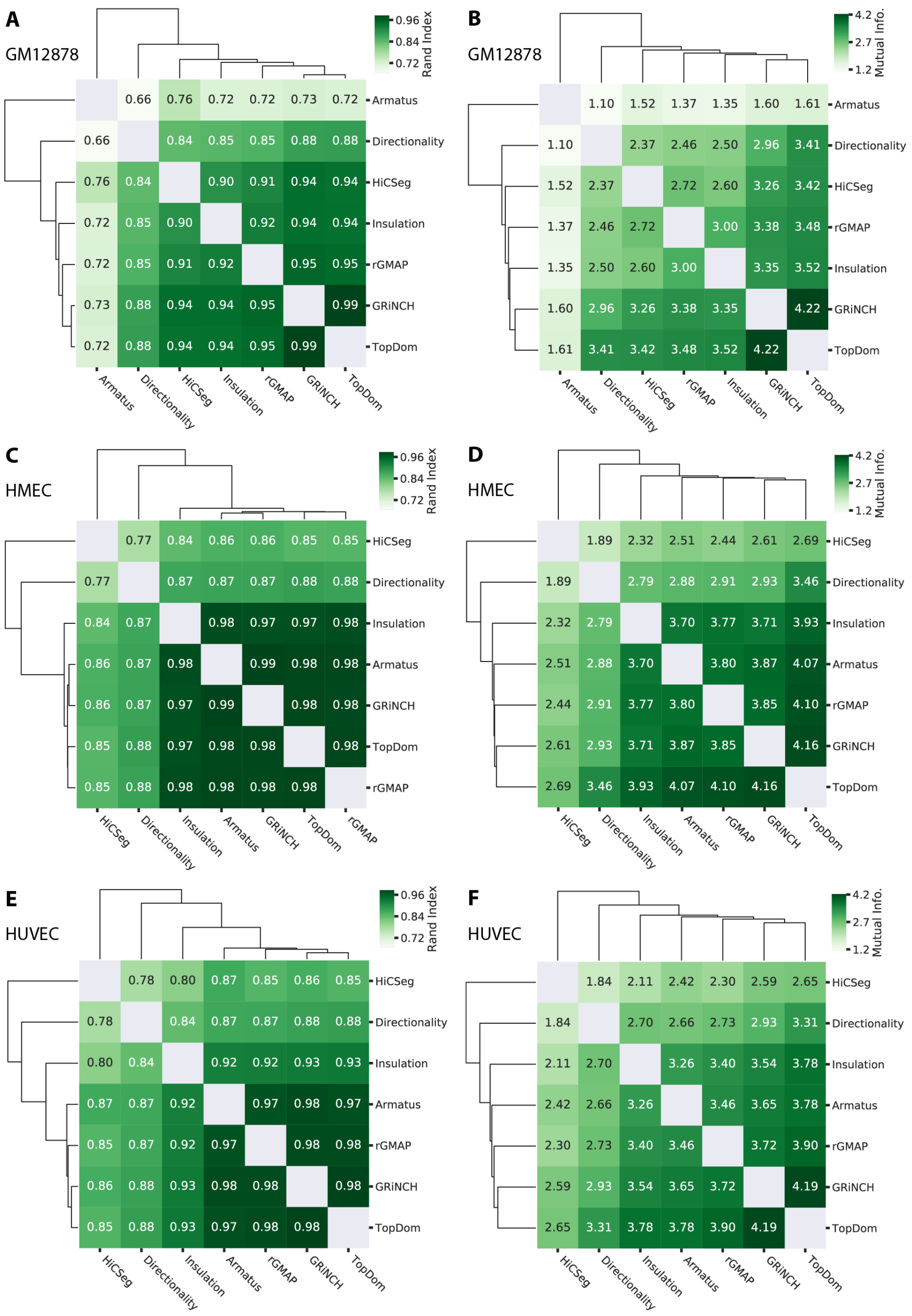

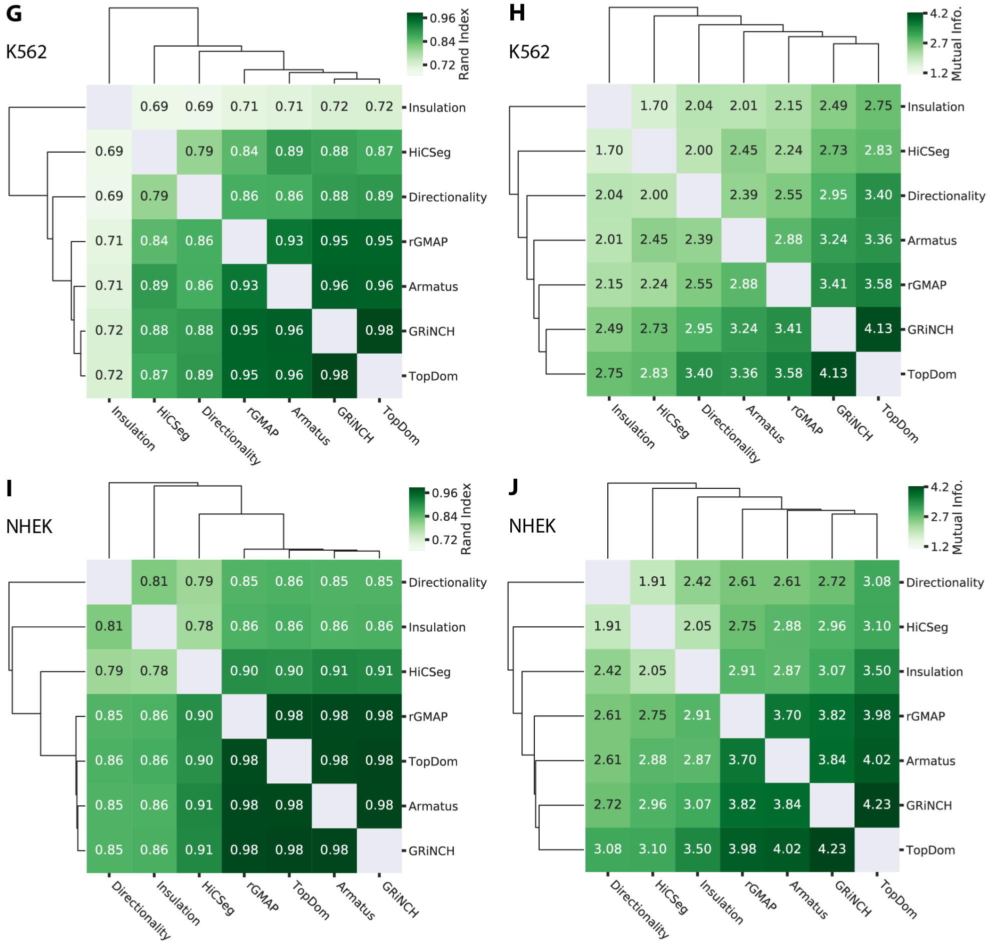

Figure S4: Evaluating similarity of TADs from different TAD-calling methods using Rand Index (**A**, **C**, **E**,**G**, **I**) and Mutual Information (**B**, **D**, **F**,**H**, **J**) for five cell lines from Rao et al., Gm12878 (**A**,**B**), HMEC (**C**,**D**), HUVEC (**E**,**F**), K562 (**G**, **H**), NHEK (**I**,**J**).

Supplementary Figure 5

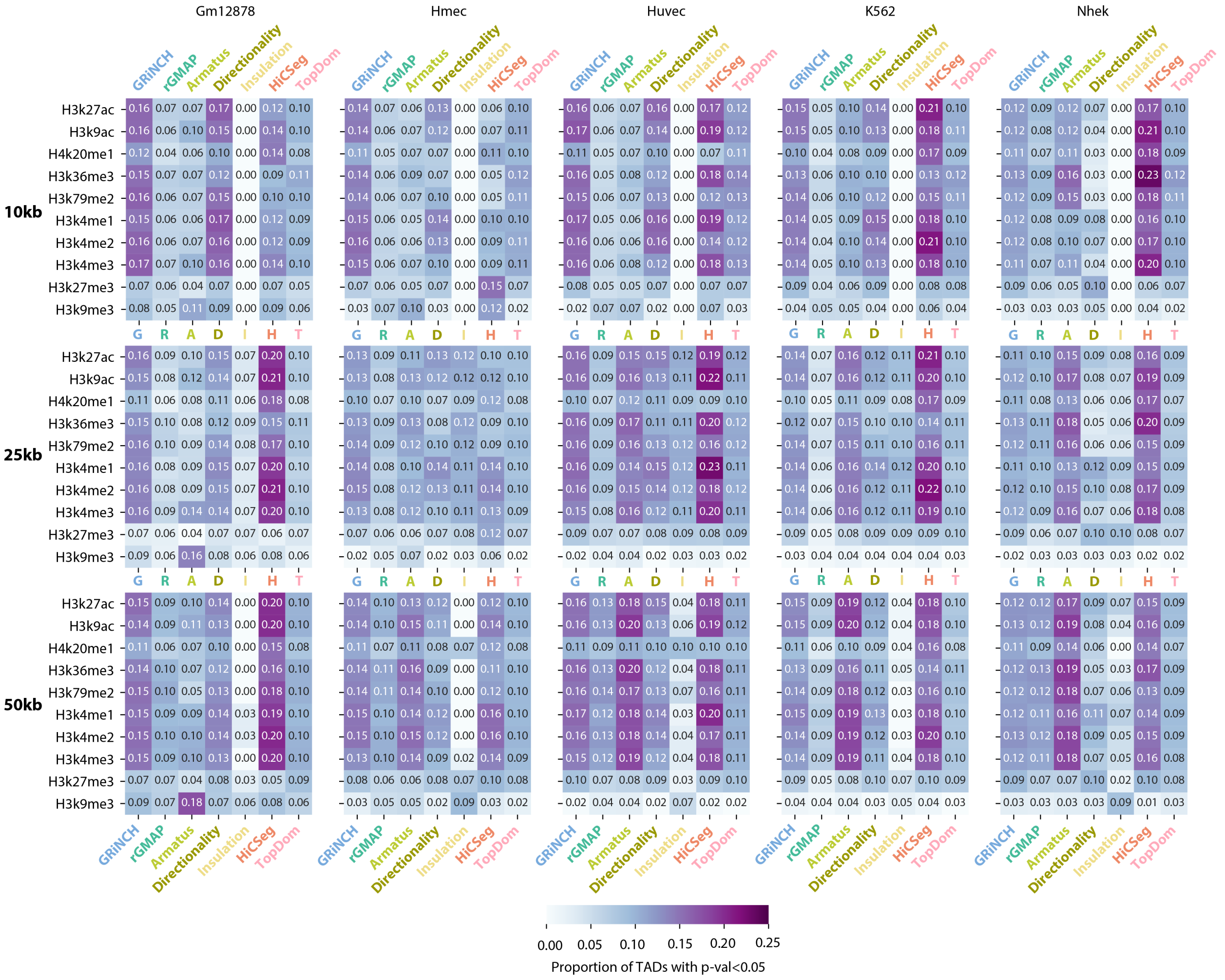

Figure S5: Proportion of TADs, from different Hi-C resolutions, with significant mean histone modification signal (i.e. empirical p-value < 0.05). The darker the entry the higher the proportion of TADs with significant histone enrichment. The average ChIP-seq signal for each histone modification mark was taken from within each TAD; the p-value of each TAD is derived from an empirical null distribution of mean signals in randomly shuffled TADs. Note: 3DNetMod outputted overlapping TADs and was excluded from this analysis as it involves TAD randomization/shuffling.

Supplementary Figure 6

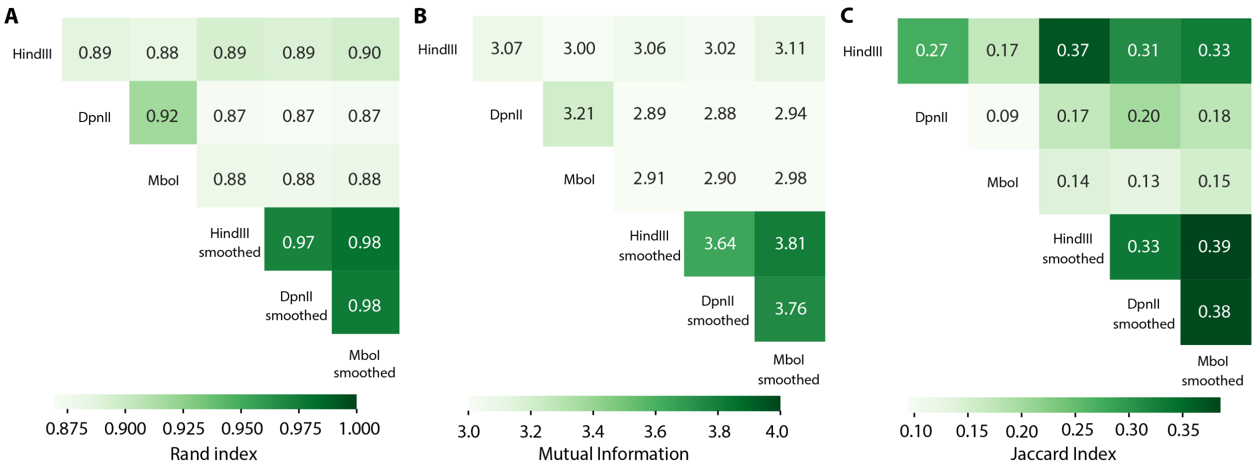

Figure S6: Similarity of structure and significant interactions among Hi-C data using different restriction enzymes and Hi-C data smoothed by GRiNCH. **A.** Similarity of Directionality TADs, measured by Rand index, from Gm12878 Hi-C data using different restriction enzymes (HindIII, DpnII, MboI) and smoothed by GRiNCH (HindIII smoothed, DpnII smoothed, MboI smoothed). **B.** Similarity of Directionality TADs, measured by mutual information. **C.** Similarity or overlap in significant interactions, measured by Jaccard Index, called by FitHiC from Gm12878 Hi-C data using different restriction enzymes (HindIII, DpnII, MboI) and smoothed by GRiNCH (HindIII smoothed, DpnII smoothed, MboI smoothed).

Supplementary Figure 7

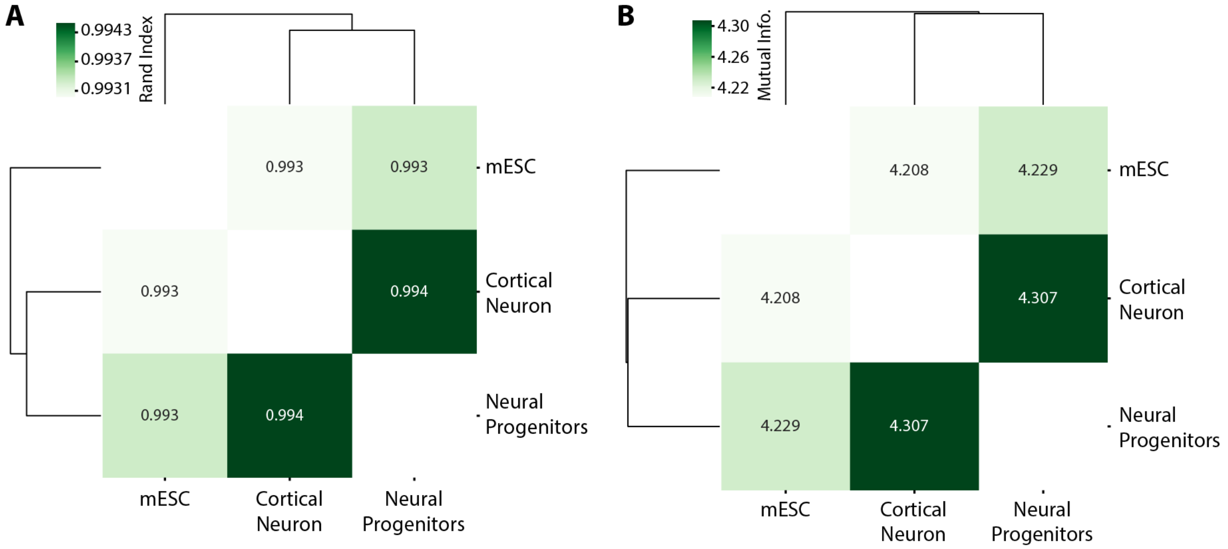

Figure S7: Similarity of GRiNCH TADs from mouse neural development time-course Hi-C data measured for three stages: mESC, Cortical Neuron, Neural Progenitors. The order of the developmental stages is mESC, Cortical Neurons, Neural Progenitors. **A**. Similarity of TADs by measured by Rand index. **B**. Similarity of TADs measured by Mutual Information.

Supplementary Figure 8

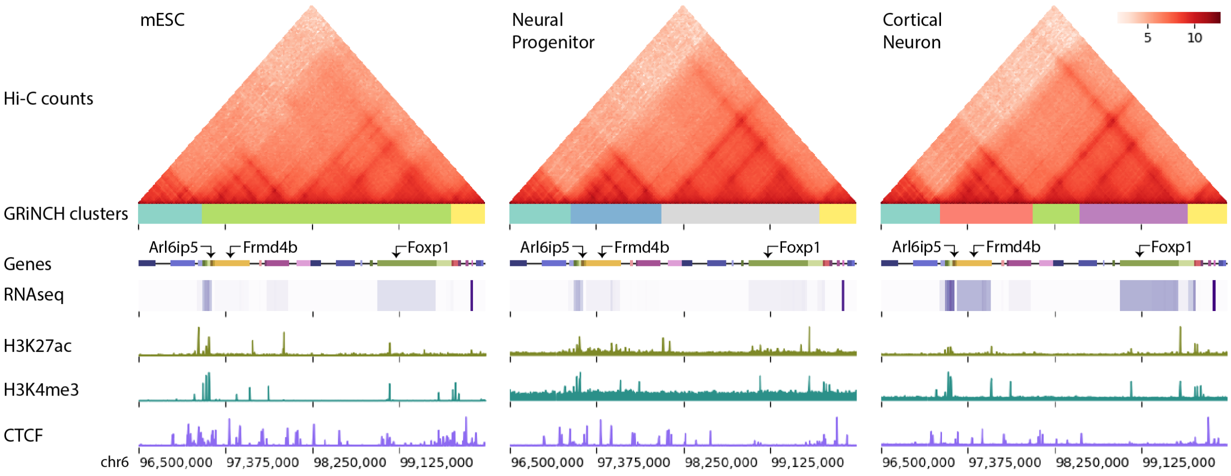

Figure S8: Interaction profile near Arl6ip5 and Foxp1 in mouse embryonic stem cells (mESC), neural progenitors (NPC), and differentiated cortinal neurons (CN). Heatmaps are of Hi-C matrices after log2-transformation of interaction counts for better visualization. GRiNCH clusters are visualized as blocks of different colors under the heatmap of interaction counts. Genes in the nearby regions are marked by small boxes, and a heatmap of their corresponding RNA-seq levels (in TPM) is shown underneath each gene. ChIP-seq signals from H3K27ac, H3K4me3, and CTCF are shown as separate tracks.

Supplementary Figure 9

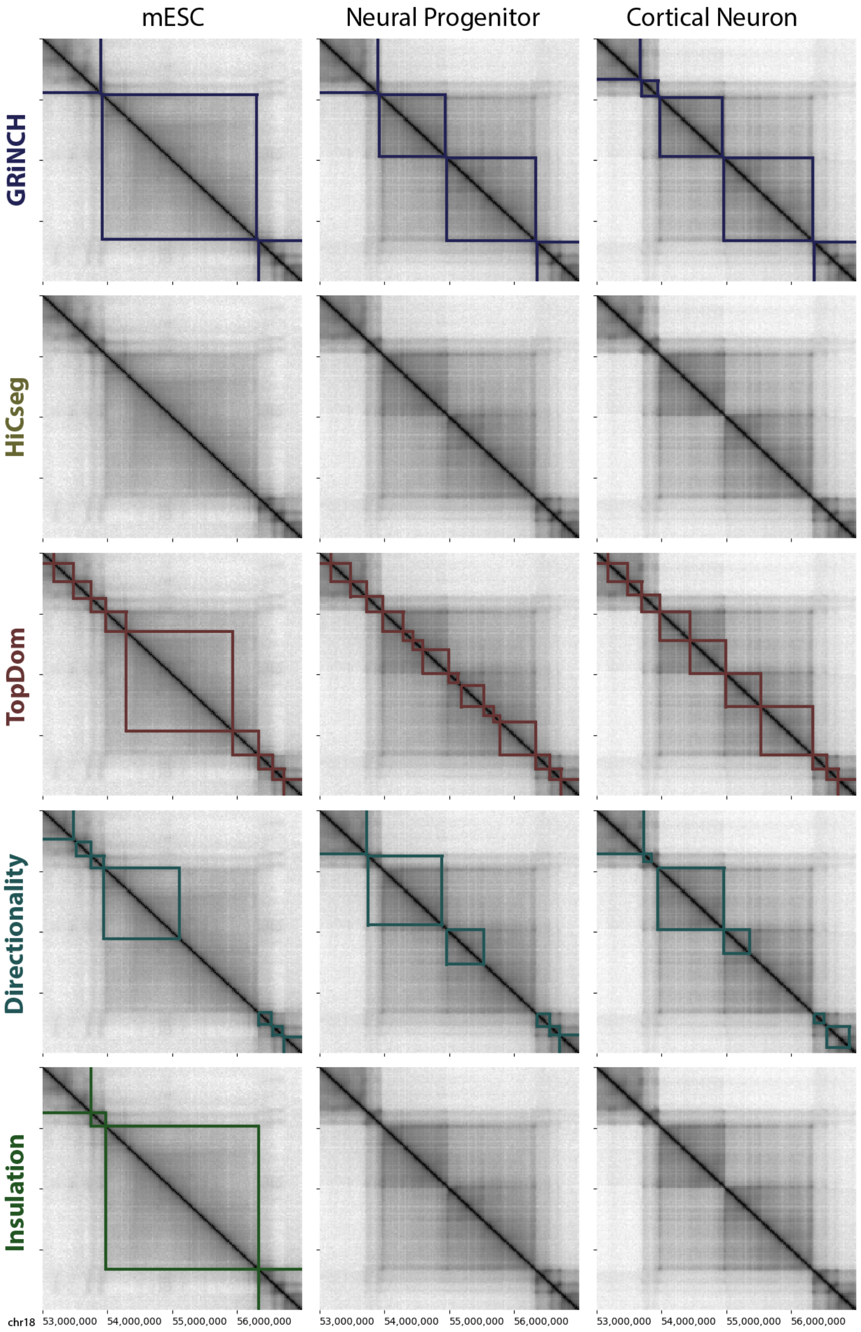

Figure S9: Visual comparison of TADs identified by different TAD-calling methods in mouse neural development time-course data. The grey heatmap visualizes the interactions surrounding Zfp608 in chr18 (same region visualized in Figure 7A in main text). Interactions counts were log2-transformed for better visualization. The boxes represent TAD boundaries.

Supplementary Figure 10

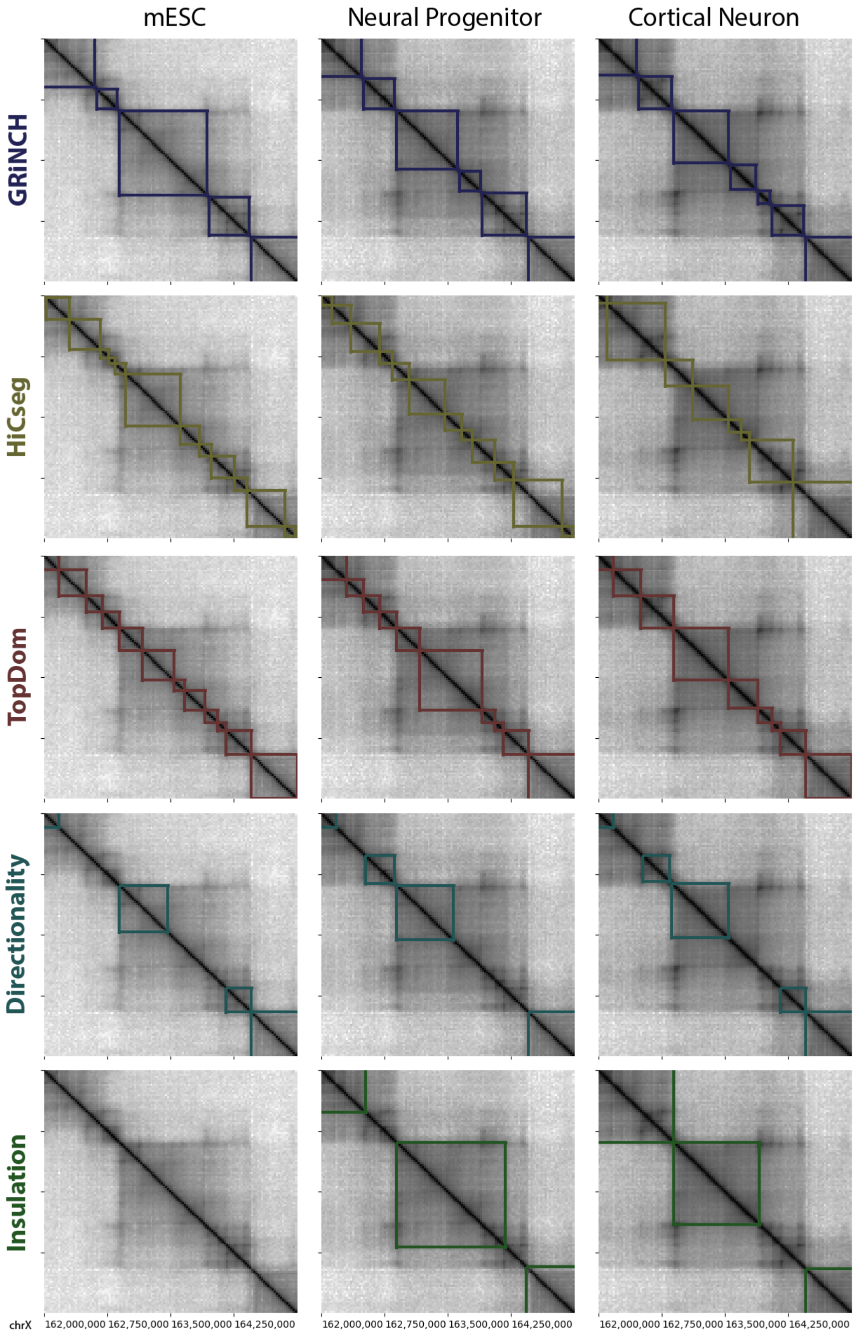

Figure S10: Visual comparison of TADs identified by different TAD-calling methods in mouse neural development time-course data. The grey heatmap visualizes the interactions surrounding Syap1 and Ap1s2 in chrX (same region visualized in Figure 7B in main text). Interactions counts were log2-transformed for better visualization. The boxes represent TAD boundaries.

Supplementary Figure 11

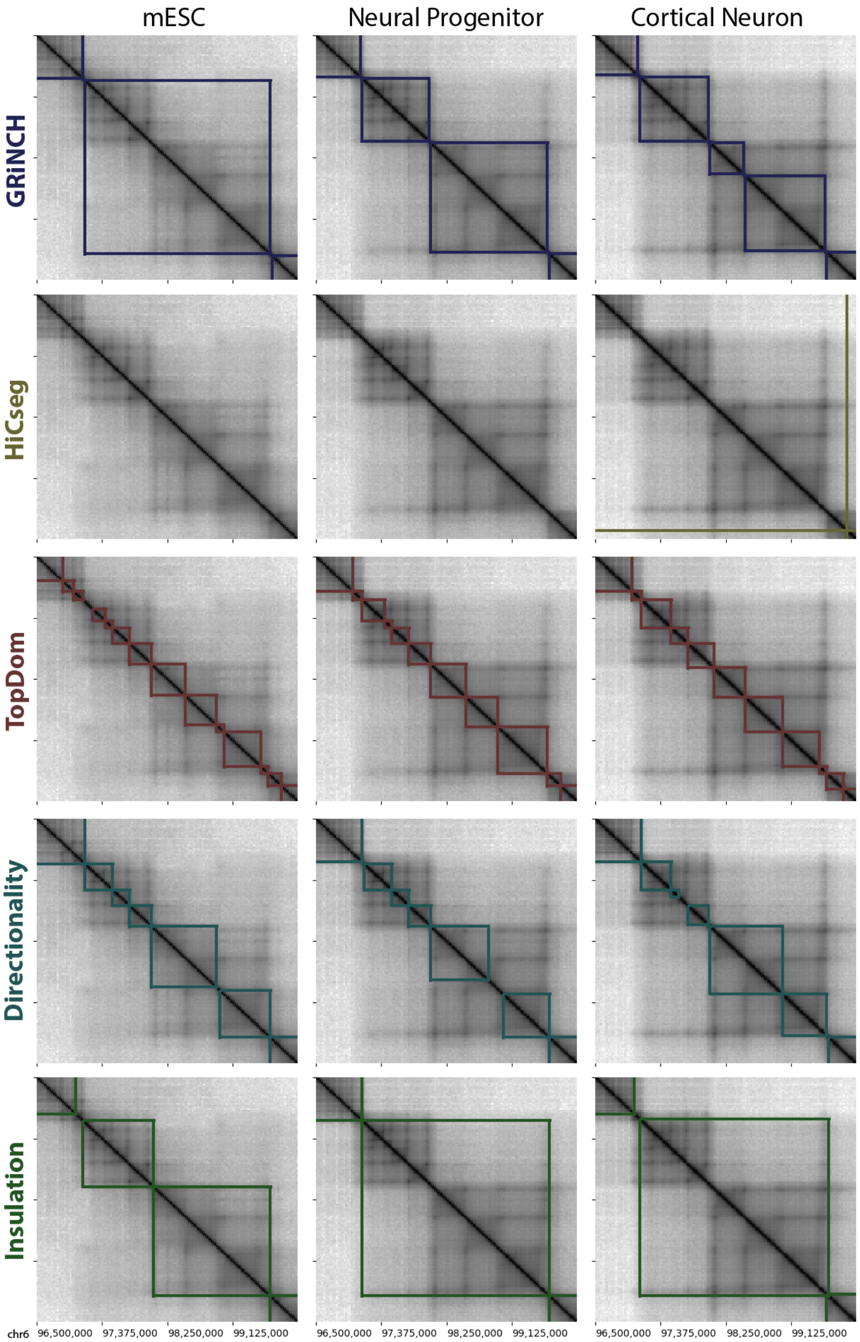

Figure S11: Visual comparison of TADs identified by different TAD-calling methods in mouse neural development time-course data. The grey heatmap visualizes the interactions surrounding Arl6ip5 and Foxp1 in chr6 (same region visualized in Figure S10 above). Interactions counts were log2-transformed for better visualization. The boxes represent TAD boundaries.

Supplementary Figure 12

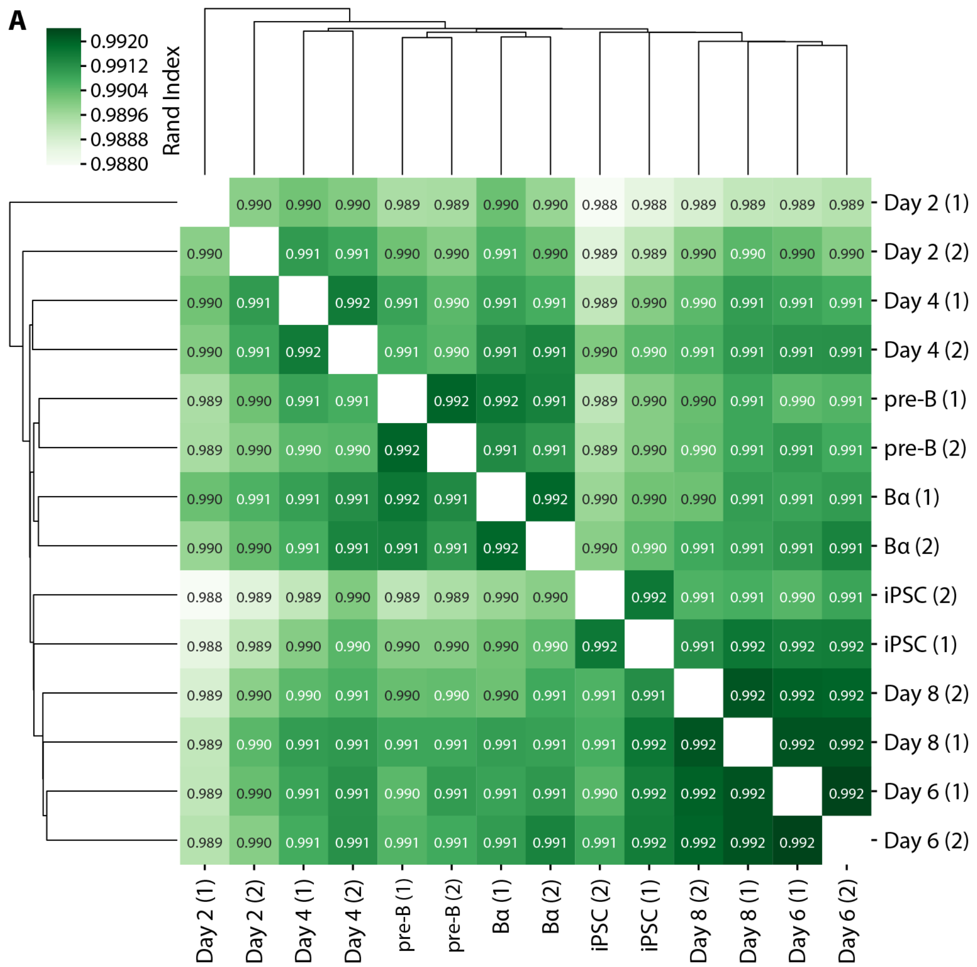

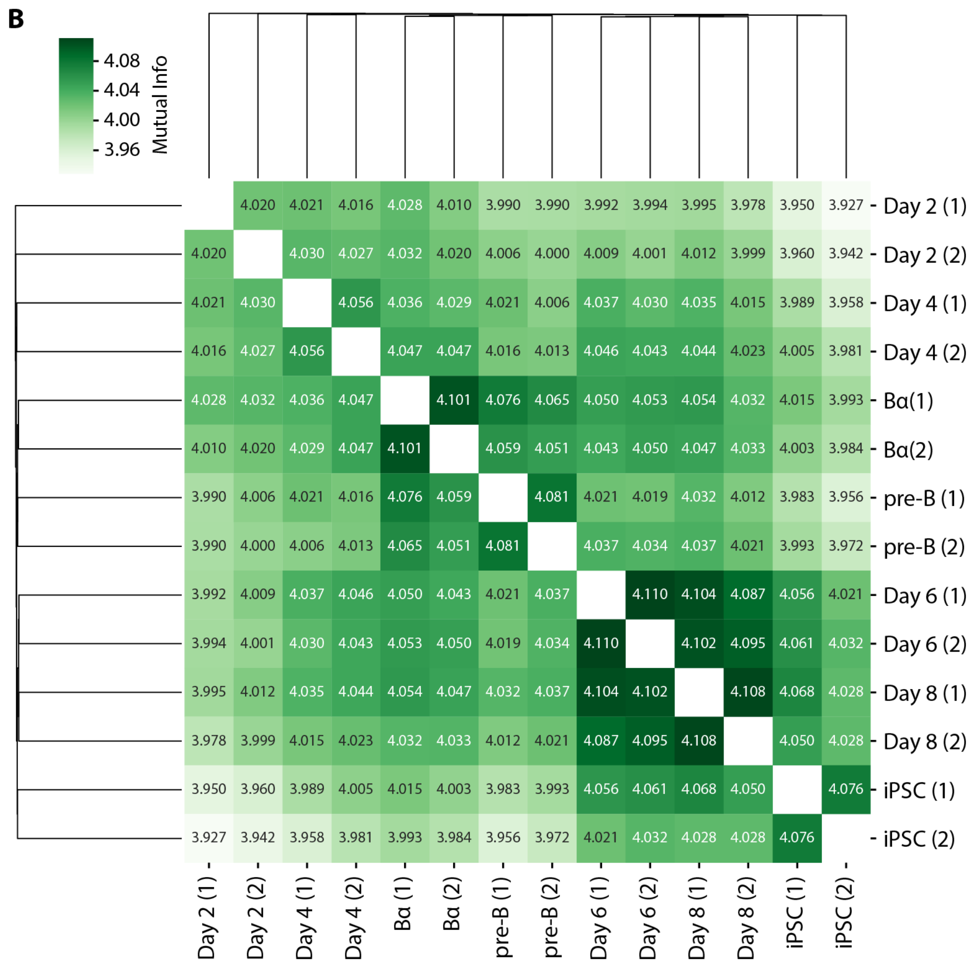

Figure S12: Similarity of GRiNCH TADs from pluripotency reprogramming time-course Hi-C data measured for the starting pre-B cells and ending induced pluripotent (iPSC) state and five intermediate time points, Bα, Day2, Day4, Day6, Day8. (1) and (2) suffixes represent replicate 1 and 2 respectively. **A**. Similarity of TADs measured by Rand index. **B**. Similarity of TADs measured by Mutual Information.

Supplementary Figure 13

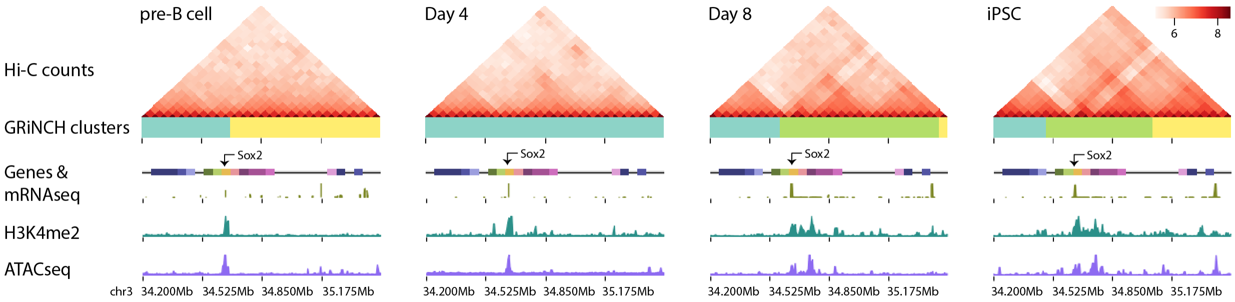

Figure S13: Interaction profile near the Sox2 gene in mouse pre-B cells, in day 4 and day 8 of reprogramming, and in induced pluripotent stem cell (iPSC). Heatmaps are of Hi-C matrices after log2-transformation of interaction counts for better visualization. GRiNCH clusters are visualized as blocks of different colors under the heatmap of interaction counts. Genes in the nearby regions are marked by small boxes, and peaks of their corresponding RNA-seq levels are shown underneath each gene. ChIP-seq signals from H3K4me2 and ATAC-seq signals are shown as separate tracks.

Supplementary Figure 14

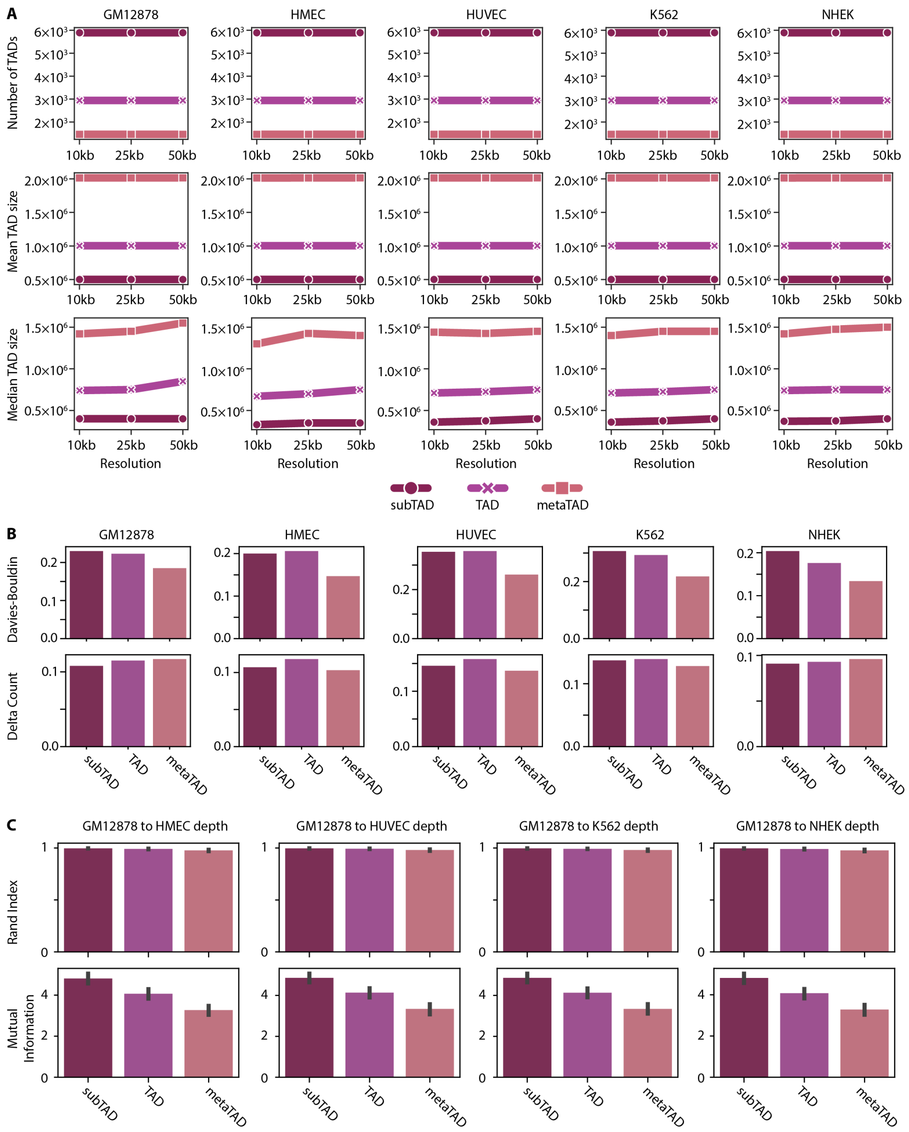

Figure S14: Characterizing GRiNCH clusters of different size scales, i.e., subTADs, TADs, metaTADs. Shown are different statistics for different settings of the number of clusters in GRiNCH, k. We set k based on the expected size of the clusters and consider three expected sizes: subTADs (500kb), TADs (1MB), metaTADs (2MB). TADs are known to be $\sim$1MB and therefore, for the "TAD scale" regions, $k = \frac{n_c}{1\text{Mb}}$, where n_c is the length of chromosome c. **A**. The number of subTADs, TADs, and metaTADs, and their median size for Hi-C datasets from five cells lines at three different resolutions: 10kb, 25kb, 50kb. **B**. Proportion of subTADs, TADs, or metaTADs with significantly better (p-val < 0.05) cluster quality metrics than random clusters, as measured by Davies-Bouldin Index and Delta Count. Results here are shown for 25kb-resolution data. **C**. The similarity between subTADs, TADs, and metaTADs from high-depth GM12878 dataset and those from datasets downsampled to other lower depths available in different cell lines. Similarity is measured by Rand Index and Mutual Information. Results here are shown for 25kb-resolution data.

Supplementary Figure 15

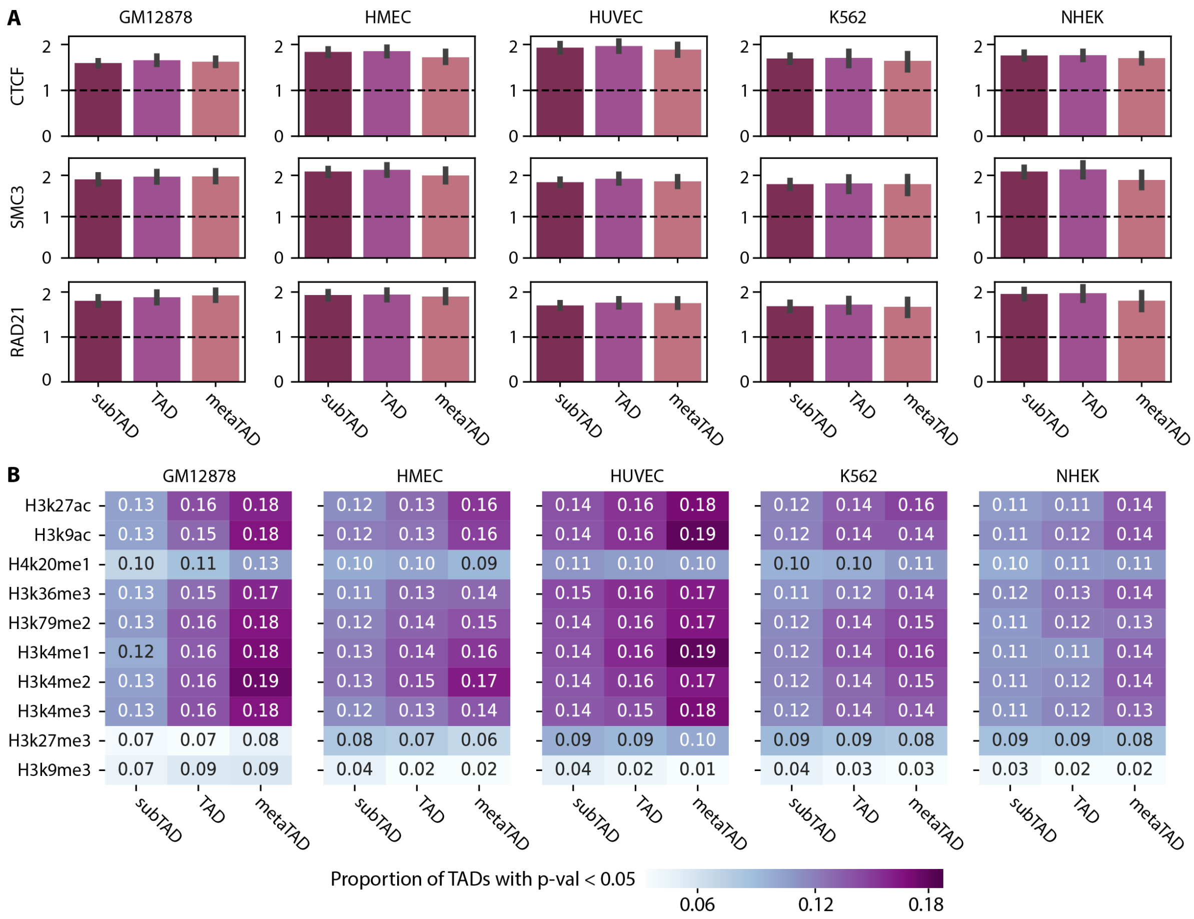

Figure S15: Enrichment of regulatory signals in GRiNCH clusters of different expected sizes: subTAD (500kb), TAD (1Mb) , and metaTAD (2Mb). The expected size is used to set the GRiNCH parameter k, the number of clusters. Results are shown for 25kb resolution data. **A**. Fold enrichment of architectural protein binding signals in subTAD, TAD, and metaTAD boundaries. **B**. Proportion of subTADs, TADs, and metaTADs with significantly higher values (p-val $<$ 0.05) of mean histone modifications compared to random clusters.

Supplementary Figure 16

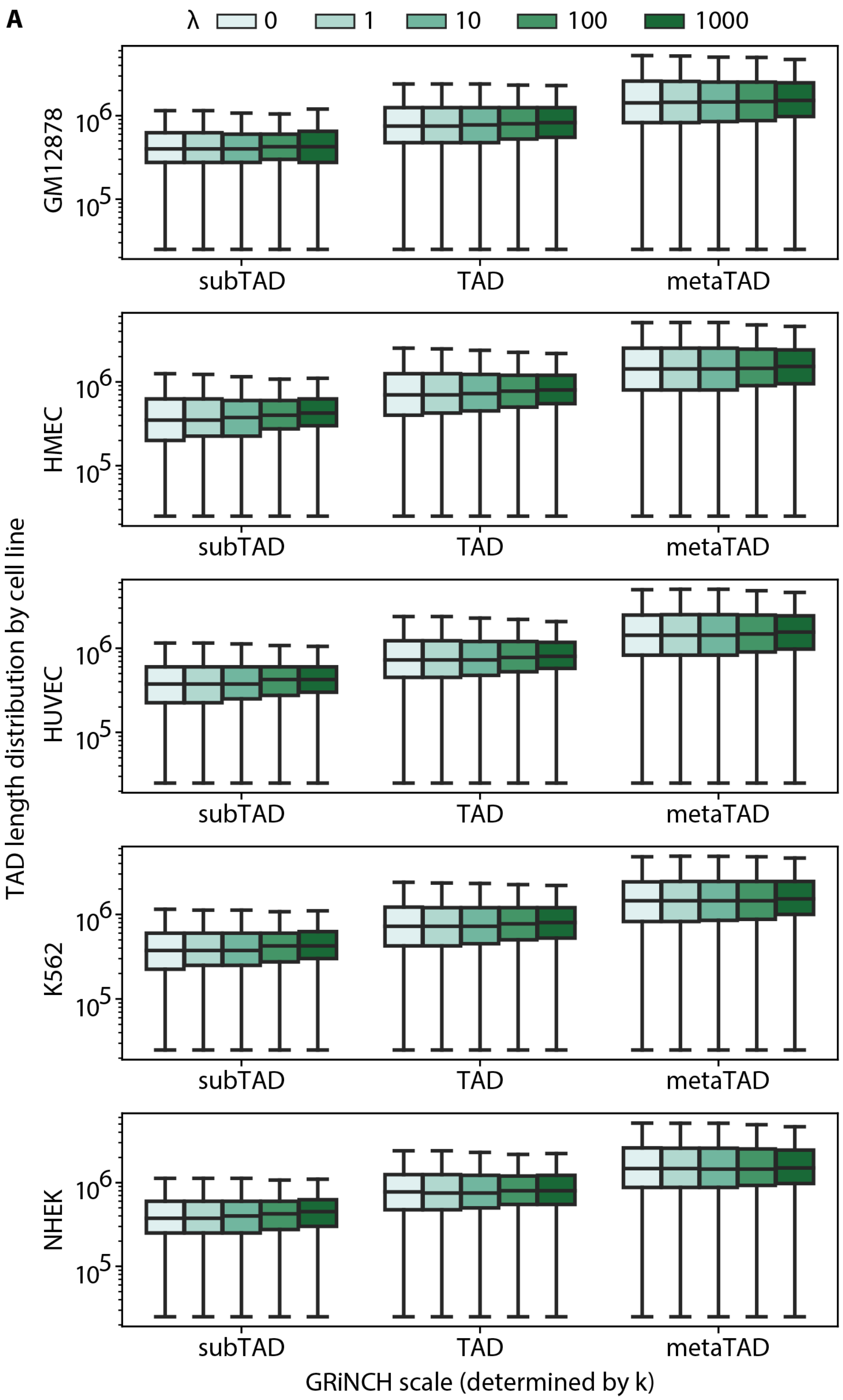

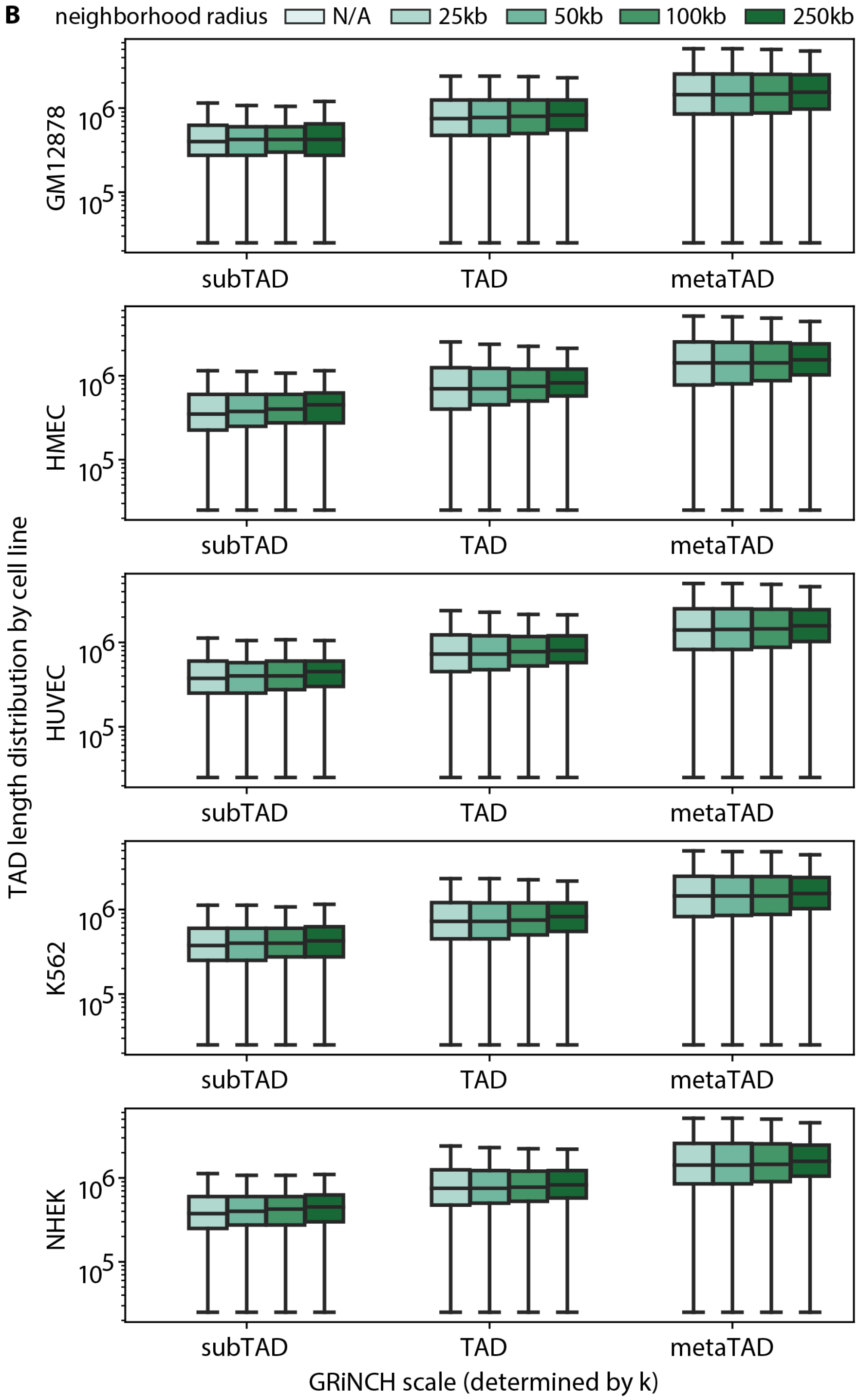

Figure S16: GRiNCH TAD size distribution by graph regularization parameters **A.** λ and **B.** neighborhood radius. $\lambda = 0$ and neighborhood radius of "N/A" correspond to no regularization. Shown are distributions for different settings of the number of clusters in GRiNCH, k. We set k based on the expected size of the clusters and consider three expected sizes: subTADs (500kb), TADs (1MB), metaTADs (2MB). TADs are known to be ~1MB and therefore, for the "TAD scale" regions, $k = \frac{n_c}{1\text{Mb}}$, where n_c is the length of chromosome c.

Supplementary Figure 17

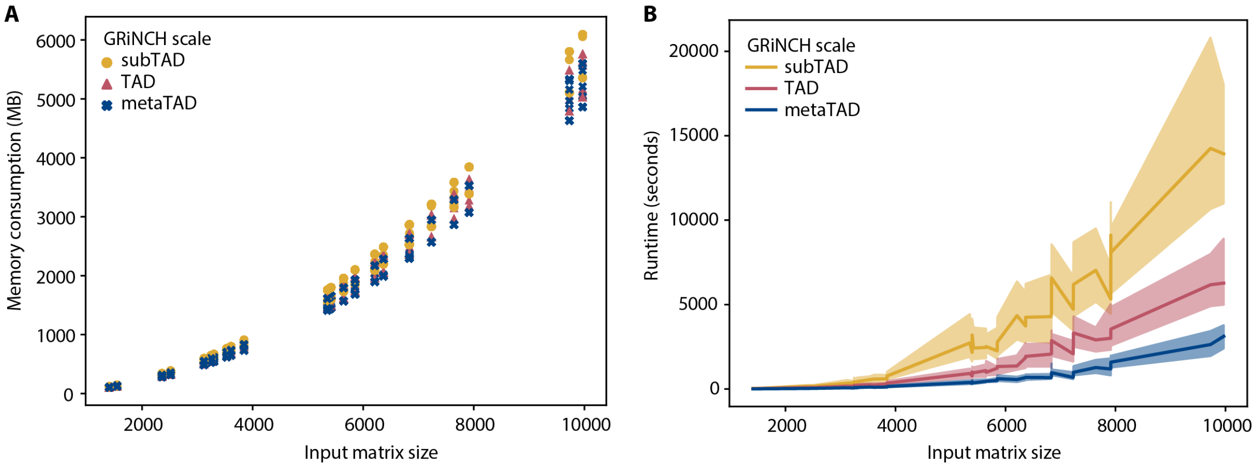

Figure S17: Memory consumption and runtime trend of the GRiNCH algorithm. **A.** Memory consumption plotted against the input Hi-C matrix size (determined by the size of the chromosome and Hi-C resolution). Each point represents the maximum resident set size for a run of GRiNCH for a given input matrix size. The GRiNCH scales (subTAD, TAD, and metaTAD) represent the chromosome-specific k parameter value, which is set based on the expected size of an output TAD/cluster (500kb, 1Mb, 2Mb respectively); for the same input matrix size, metaTAD setting uses k value that is half of the TAD setting and TAD uses k half of subTAD setting. For a given matrix size and k combination, GRiNCH was run with every combination of regularization parameters $\lambda \in \{1, 10, 100, 100\}$ and neighborhood radius $\in$ { 25kb, 50kb, 100kb, 250kb, 500kb, 1Mb } as well as without any regularization ($\lambda = 0$). These runs were completed across a distributed computing platform with machines of varying computing power. **B.** Runtime distribution of GRiNCH against the input Hi-C matrix size. GRiNCH runtime was measured for different matrix sizes, TAD scales, and regularization parameters (see **A**), from start to successful termination of program. The dark line in the middle represents the median runtime of GRiNCH. The top of the shaded area represents the 75th percentile, the bottom 25th percentile of runtimes.

Supplementary Figure 18

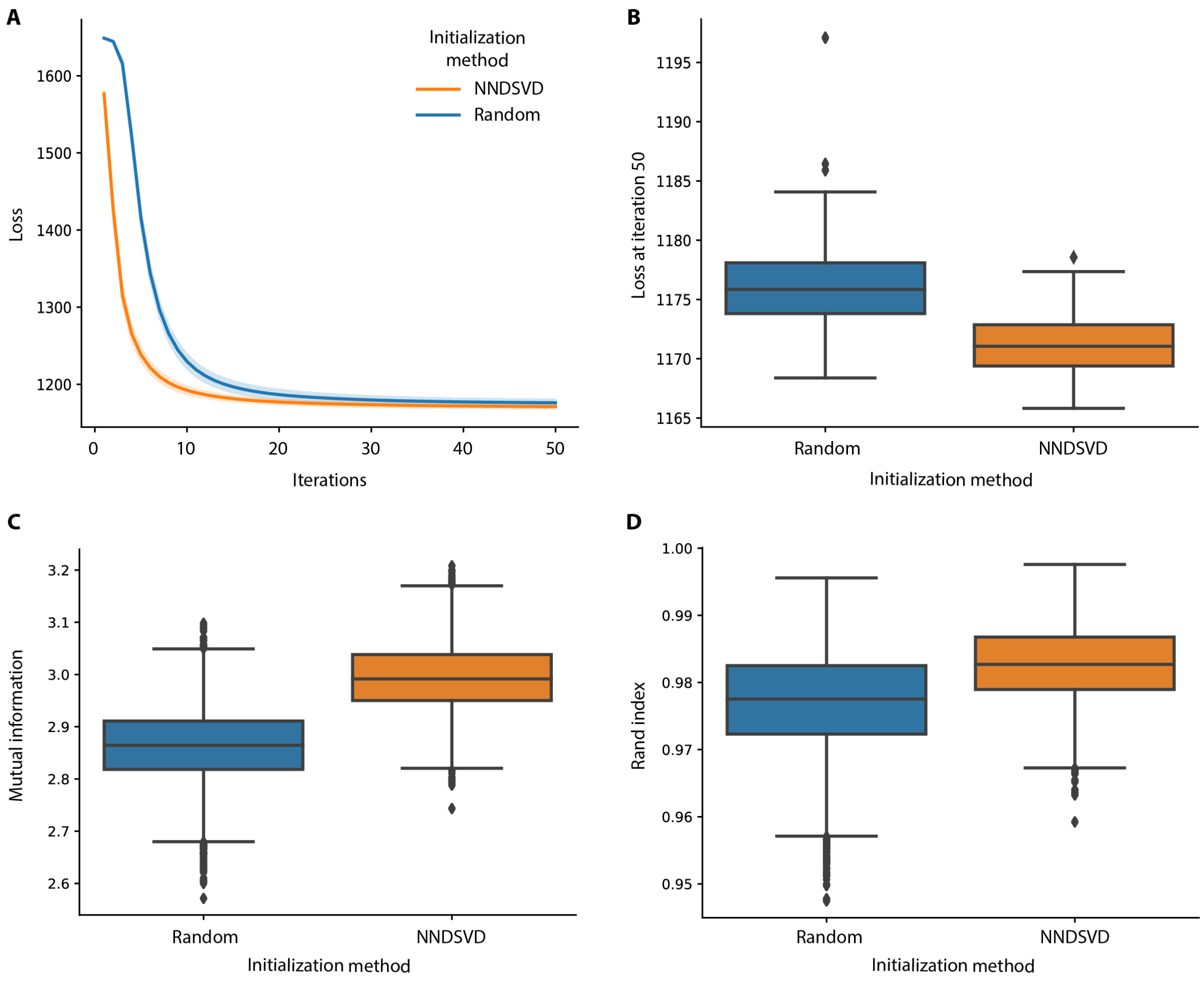

Figure S18: Comparison of NNDSVD initialization versus random initialization. **A**. Loss as a function of iterations of the GRiNCH algorithm with NNDSVD initialization versus random initialization. For each type of initialization, we tracked the value of the objective/loss over 50 iterations of the GRiNCH algorithm, using 100 different seeds for randomization. The solid line represent the mean loss at a given iteration, and the lightly colored bands around each line is the standard deviation of the loss at the given iteration. **B**. The distribution of the loss after 50 iterations for each type of initialization. Each point on the box plot corresponds to one of 100 different random seeds. **C**. Measuring stability of GRiNCH TADs from NNDSVD initialization versus random initialization. Each box plot shows the distribution of similarity score of GRiNCH TADs using different initialization methods. GRiNCH TADs after 50 iterations from each run with different seeds and initialization methods were converted to clusters. Pairwise similarity of clustering results from seed x and seed y using the same initialization method was measured with mutual information. Higher mutual information means the GRiNCH TADs tend to be more similar and therefore more stable across different seeds. **D**. Measuring stability of GRiNCH TADs with Rand Index. GRiNCH TADs were generated in the same as in **C**. Higher Rand index means the GRiNCH TADs tend to be more similar and therefore more stable across different seeds.

List of Supplementary Tables

Table S1(A) Ranking TAD-calling methods by the percentage of TADs with significant Davies-Bouldin Index across 5 cell lines

Table S1(B) Ranking TAD-calling methods by the percentage of TADs with significant Delta Contact Count across 5 cell lines

Table S1(C) Ranking TAD-calling methods by mean (absolute) change in median TAD size as input data resolution changes from 10kb to 25kb to 50kb

Table S1(D) Ranking TAD-calling methods by mean Rand Index between TADs from two different resolutions of Hi-C data.

Table S1(E) Ranking TAD-calling methods by mean Mutual Information between TADs from two different resolutions of Hi-C data.

Table S1(F) Ranking TAD-calling methods by mean Rand Index between TADs from high-depth Gm12878 data and TADs from Gm12878 data downsampled to other cell lines' depth.

Table S1(G) Ranking TAD-calling methods by mean mutual information between TADs from high-depth Gm12878 data and TADs from Gm12878 data downsampled to other cell lines' depth.

Table S1(H) Ranking TAD-calling methods by mean fold enrichment of known boundary elements (CTCF, SMC3, RAD21) across 5 cell lines.

Table S1(I) Ranking TAD-calling methods by mean proportion of TADs with significant histone modification signals across 5 cell lines

Table S2 Ranking of transcription factors by significant motif enrichment in TAD boundaries across all cell types or time points during mouse pluripotency reprogramming (mouse pre-B cell, D2, D4, D6, D8, iPSC)

Table S3 Ranking of transcription factors by significant motif enrichment in TAD boundaries across all cell lines (GM12878, HUVEC, HMEC, NHEK, K562) from Rao et al.
