# Supplementary material for: GRiNCH: simultaneous smoothing and detection of topological units of genome organization from sparse chromatin contact count matrices with matrix factorization
Source: Genome Biol. 2021 May 25;22:164. doi: 10.1186/s13059-021-02378-z (PMC8152090; doi:10.1186/s13059-021-02378-z)
Supplement: Supplementary file 2 — Additional file 2 Algorithm.pdf. A PDF file containing Algorithm 1, the pseudocode for chain-constrained k-medoids clustering. [file 13059_2021_2378_MOESM2_ESM.pdf]

Algorithm 1: Chain-constrained k-medoids clustering

Input: U $\in \mathbb{R}^{n \times k}$, one of the factors from NMF, and $maxIter$, the maximum number of iterations

Output: The cluster assignments, $\mathcal{C} \in \{c_1, c_2, \ldots, c_n\}$, for each of the chromosomal bins

Initialize k medoids to be the rows with the largest value from each column of U

Initialize an empty priority queue Q

while *numIter* $<$ *maxIter* **do**

    Add current medoids to priority queue Q, with priority value of 0

    // Q orders bins by ascending priority values.

    while Q *is not empty* **do**

        Pop bin b from Q

        if b *is not assigned to a cluster yet* **then**

            /* First, assign bin to cluster */

            if b *is a medoid* **then**

                Assign b to its own cluster

            else

                Assign b to either: the same cluster as its nearest upstream neighbor along the chromosome already assigned to a cluster, u, or the same cluster as its nearest downstream neighbor along the chromosome already assigned to a cluster, d, based on the similarity between the latent feature vectors of b and the cluster medoids, i.e., $\min_{c \in \{u,d\}} \|U[b,:] - U[\text{medoid of } c,:]\|$

            /* Next, add any unassigned neighbor to priority queue: */

            for *each immediate upstream or downstream neighbor i of b not assigned to a cluster* **do**

                Add i to Q with priority $=$ priority of $b + \|U[b,:] - U[i,:]\|$

    Update medoids

    if *sum of distances between each bin and its cluster medoid didn't change from last iteration* **then**

        Break
