# Supplementary material for: GRiNCH: simultaneous smoothing and detection of topological units of genome organization from sparse chromatin contact count matrices with matrix factorization
Source: Genome Biol. 2021 May 25;22:164. doi: 10.1186/s13059-021-02378-z (PMC8152090; doi:10.1186/s13059-021-02378-z)
Supplement: Supplementary file 4 — Additional file 4 Review history [file 13059_2021_2378_MOESM4_ESM.docx]

Review History

**First round of review**

**Reviewer 1**

**Were you able to assess all statistics in the manuscript, including the appropriateness of statistical tests used?**

Yes N/A

**Were you able to directly test the methods?**

Yes

**Comments to author:**

The main contribution of this work is that they developed a smoothing method to identify TADs from sparse contact count matrices. The results are quite stable at different resolutions of Hi-C maps, especially at low sequencing depth. Below are my suggestions to improve the clarity and readability of this study.

1.Line 49, Page 3. "five different cell lines" - The smoothing method might be affected by different fragment distribution, induced by the restriction enzymes used in the study (DpnII, HindIII, Arima). Can authors check whether their method can be used for Hi-C data generated by different cutters? Or even more challenging, 6 base cutters vs 4 base cutters.

2. Line 62, Page 3. The results will be more convincing if the author can compare the GRiNCH results with others by using different portion of TADs. For example, 50%, 75%, 90% …

3. Line 16, Page 4. The presented median length of TADs is reasonable, but it might be better if they can provide a violin plot.

4. Line 28, Page 5. 1. I think the contributions of topdom, rGMAP, and 3DNetMod is that they can hierarchical subTADs. As a result, their CTCF enrichment score is not that high. The authors can discuss this point.

5. Line 38, Page 5. Clarify how the percentage of histone mod were calculated. Why the 3dnetmod were not listed here? Further, the authors should try different resolution for this analysis.

8.Line 17, Page 8. The same Mutual score and Rand index score should be applied in SPRITE and Hi-ChIP. If not, the authors need to explain why.

**Reviewer 2**

**Were you able to assess all statistics in the manuscript, including the appropriateness of statistical tests used?**

Yes: Very solid all around

**Were you able to directly test the methods?**

No

**Comments to author:**

The authors demonstrate that the proposed approach has some advantage over existing TAD-prediction approaches, in terms of the combined quality of the predictions and robustness. The authors go on to demonstrate that their TAD predictions are enriched for the expected DNA-binding protein occupancy and histone modifications; their results show that GRINCH is comparable or superior to competing approaches. Overall, the manuscript introduces a welcome addition to the existing set of TAD-prediction tools, and it is a model in terms of assessment its accuracy and robustness. Yet it lacks the kind of biological insights that are typical of papers published in this journal. What insights can one obtain from using the proposed tool rather than some of the other existing tools? On this question, I feel that the manuscript falls short.

Major comments:

1) The authors go on to analyze Bonev's neural differentiation HiC data and a dataset on reprogramming of mouse pre-B cells to pluripotent stem cells. The analyses performed here are not very exciting or new, and are merely an illustration of the use of their tool. Would one have obtained very different results if they had used another TAD predictor? The type of motif analyses reported here have been done before - what are we learning that is new? How is this attributable to the quality of GRINCH?

2) Overlapping TADs, and TADs with multiple possible boundaries cause difficulties to all TAD predictors. How does GRINCH behave in these situations?

3) The analysis of robustness of predictions against different resolutions is important. The authors focus on the stability of predicted TAD length distribution as a measure of robustness; it would be useful to compare the predictions themselves (i.e. do the same TADs get predicted, plus or minus a fudge factor to account for varying resolution?). Also, since the number of TADs predicted is strongly determined by the choice of hyper-parameters, rather than by resolution, isn't this focus on the stability of the number of TADs predicted a bit unfair?

4) The smoothing properties of GRINCH, and its ability to detect significant interactions, should be compared against some of the many existing tools that already exist to do so.

5) The smoothness weight lambda and dimensionality k probably has some consequences on the size distribution of predicted TADs. Is this the case? How strongly is the number of predicted TADs determined by k?

6) How does the approach compare conceptually to Hu et al. 2016 ( https://www.ncbi.nlm.nih.gov/pmc/articles/PMC4908340/ ), which is also based on non-negative matrix decomposition?

7) It would be useful to discuss the running time and memory requirement of GRINCH, especially at higher resolution.

Minor comments:

1) What do you mean by randomly shuffle TADs?

2) "we first took the highest-depth dataset (GM12878, 86 million reads total)". This seems like a very low coverage data set? Is this only for one chromosome?

**Authors Response**

**Point-by-point responses to the reviewers’ comments:**

Reviewer #1:

The main contribution of this work is that they developed a smoothing method to identify TADs from sparse contact count matrices. The results are quite stable at different resolutions of Hi-C maps, especially at low sequencing depth. Below are my suggestions to improve the clarity and readability of this study.

*We thank the reviewer for this comment and the suggestions below.*

1.Line 49, Page 3. "five different cell lines" - The smoothing method might be affected by different fragment distribution, induced by the restriction enzymes used in the study (DpnII, HindIII, Arima). Can authors check whether their method can be used for Hi-C data generated by different cutters? Or even more challenging, 6 base cutters vs 4 base cutters.

*We have performed additional analysis with GM12878 25kb Hi-C data from three different protocols: (1) in situ Hi-C using MboI for digestion, (2) in situ Hi-C using DpnII, and (3) dilution Hi-C using HindIII (lines 622-625 in Methods). We smoothed the three datasets using GRiNCH, then measured the concordance of structure (or agreement among sets of TADs) and overlap in significant interactions. After smoothing, we called TADs on the original and the smoothed data using an independent TAD-calling method (Directionality Index). The agreement or similarity of TADs, measured by Rand Index and Mutual Information, were much higher among the datasets smoothed by GRiNCH (Figure S6A, B). Furthermore, we measured the overlap in the sets of significant interactions from different datasets with Jaccard Index (lines 951-956 in Methods). We again find that GRiNCH-smoothed data shared a larger portion of significant interactions among them (Figure S6C). We report these results, which suggest GRiNCH smoothing helps recapitulate the structure and significant interactions from data with shared underlying biology, in lines 266-286.*

2. Line 62, Page 3. The results will be more convincing if the author can compare the GRiNCH results with others by using different portion of TADs. For example, 50%, 75%, 90% ...

*Thank you for bringing to attention the need to clarify this evaluation procedure. The reviewer is referring to the following statement: “For each TAD-finding algorithm, we estimated the proportion of TADs with significantly better DBI or DCC value than randomly shuffled TADs”. We believe the reviewer is asking how the proportion of significant GRiNCH TADs compares to other methods. This is what we did, however, our procedure for generating random TADs (which also pertains to another comment on the histone modification enrichment analysis below) was not clear. We clarified the purpose of and the procedure for generating the random TADs in “Methods - Internal validation metrics” (lines 784-788, 797-805, 813-818), and direct the readers to the Methods section in the Results (line 114-117). Briefly, in order to assess whether the internal validation metric value (Davies-Bouldin Index, or DBI, and Delta Contact Count, or DCC) calculated for an individual TAD is of significance (rather than an artifact of a few close-by interactions), we generated a background/null distribution of DBI and DCC values from randomly shuffled TADs. Random shuffling of TADs entails taking all “real” TADs and any non-TAD gaps between them, without any sampling, and randomly moving them within the chromosome to preserve TAD length distribution within each method (procedure detailed in lines 762-767). Against this null distribution collected from randomized TADs, we determine the empirical p-value for a “real” TAD and its significance; we then calculate, for each method, the proportion of “real” TADs with significant DBI or DCC values.*

*When we were reassessing our results we found that the non-TADs for some methods (Directionality, Insulation) were not analyzed correctly. We have addressed this issue and see an improved proportion for these methods (Figure 2A). However, the overall trends remain the same.*

3.	Line 16, Page 4. The presented median length of TADs is reasonable, but it might be better if they can provide a violin plot.

*Thank you for this suggestion. We have made the following updates: (1) updated Figure 2B with the violin plot of TAD length distribution from GM12878 cell line, (2) included the visualization for the rest of the cell lines (HMEC, HUVEC, K562, and NHEK) as a supplementary figure (Figure S2), and (3) reference the figures in lines 129 – 130 in Results.*

4.	Line 28, Page 5. 1. I think the contributions of topdom, rGMAP, and 3DNetMod is that they can hierarchical subTADs. As a result, their CTCF enrichment score is not that high. The authors can discuss this point.

*Thank you for the comment. We wanted to clarify that in the original version of the paper we had mentioned Armatus, rGMAP and 3DNetmod at this location. We clarified in Methods that Armatus provides a set of non-overlapping consensus TADs, consolidated from the initial set of overlapping TADs (lines 700-701), and rGMAP provides both a list of hierarchical domains and a list of non-overlapping TADs (lines 723-724). We used non-overlapping TADs in both cases. Although we used the non-overlapping TADs for benchmarking when available (i.e. for Armatus and rGMAP), these approaches might be tuned towards hierarchical domain discovery, resulting in lower CTCF enrichment. We have updated the Results (lines 194-195) to reflect this point.*

5.	Line 38, Page 5. Clarify how the percentage of histone mod were calculated. Why the 3dnetmod were not listed here? Further, the authors should try different resolution for this analysis.

*We clarified the TAD randomization, null distribution generation, and significance calculation procedure for histone modification enrichment in lines 756-778 under Methods, and refer the readers to the Methods section from “GRiNCH TADs are enriched in architectural proteins and histone modification signals” line 199 in Results. Briefly, for each method we estimated a null distribution of histone modification signals in a TAD by generating a set of random TADs that had the same length distribution as the original TADs. Next, we asked what proportion of TADs from a method had a significantly higher value of histone modification compared to the random TADs, where significance was determined by an empirical p-value of 0.05. As 3DNetMod outputted overlapping TADs, it was excluded from any analysis involving TAD randomization and shuffling due to the nature of the shuffling procedure detailed in lines 762-767 – we noted this in Methods (lines 714-716), as well as in captions of figures reporting results where 3DNetMod was excluded (Figures 2,3,5). Finally, we have included the same histone enrichment analysis for 10kb and 50kb resolution data, in addition to the original 25kb data analysis, and find that our conclusions are consistent across resolutions (Figure S5, also noted in Results, line 204-205).*

8.Line 17, Page 8. The same Mutual score and Rand index score should be applied in SPRITE and Hi-ChIP. If not, the authors need to explain why.

*For clarity, the reviewer is referring to text near these statements in the original text: “A visual comparison between these datasets for an 8Mb region of chr8 shows regions of good concordance between datasets (Figure 8A-D). We quantified the global similarity of GRiNCH TADs from the four different datasets, for all chromosomes, with Rand Index and Mutual Information (Figure 8E,F).” As noted in the second sentence, we did compare every pair of 3D genome datasets including SPRITE and HiChIP using Rand Index and Mutual Information. Figure 8E and F show these pairwise comparisons in heatmaps.*

Reviewer #2:

The authors demonstrate that the proposed approach has some advantage over existing TAD-prediction approaches, in terms of the combined quality of the predictions and robustness. The authors go on to demonstrate that their TAD predictions are enriched for the expected DNA-binding protein occupancy and histone modifications; their results show that GRINCH is comparable or superior to competing approaches. Overall, the manuscript introduces a welcome addition to the existing set of TAD-prediction tools, and it is a model in terms of assessment its accuracy and robustness. Yet it lacks the kind of biological insights that are typical of papers published in this journal. What insights can one obtain from using the proposed tool rather than some of the other existing tools? On this question, I feel that the manuscript falls short.

*Thank you for these comments – we address the reviewer’s concern along with the first major comment below.*

Major comments:

1) The authors go on to analyze Bonev's neural differentiation HiC data and a dataset on reprogramming of mouse pre-B cells to pluripotent stem cells. The analyses performed here are not very exciting or new, and are merely an illustration of the use of their tool. Would one have obtained very different results if they had used another TAD predictor? The type of motif analyses reported here have been done before - what are we learning that is new? How is this attributable to the quality of GRINCH?

*Thank you for this comment. We had initially focused our analysis of these datasets to demonstrate a concrete use case of our method beyond summary metrics and to show that it can replicate key biological findings from previous studies. We have now systematically examined all GRiNCH TADs from Bonev et al.’s neural development data and identified two novel sites of dynamics of chromosomal organization accompanied by expression level changes in genes involved in motor control and defect, X-linked neural developmental disease, neurotransmitter transport, and neural differentiation (updated Figure 7B, Figure S8, lines 309¬327 in Results, lines 870-888 in Methods). We further visualized how other top-performing methods behave in these regions (Figures S9-11); however, we did not recover TADs and their dynamics from these results. While different hyperparameter settings could yield meaningful solutions when using these methods, we ran GRiNCH with default parameter settings. Finally, our motivation for the motif enrichment analysis was to discover additional regulatory proteins that could play a role in the dynamic TAD organization. While existing work of motif analysis in TAD boundaries has focused on a single time point or stage, to our knowledge, a cell-type-specific, accessibility-based motif enrichment analysis on topological domain boundaries using an extensive catalogue of motifs has not been reported before. We have updated our text (lines 340-345, Results) to cite these previous analyses to provide context and the biological insights we gained by applying GRiNCH.*

2)	Overlapping TADs, and TADs with multiple possible boundaries cause difficulties to all TAD predictors. How does GRINCH behave in these situations?

*GRiNCH does not produce overlapping TADs and in its default setting does not produce multiple possible boundaries for the same dataset. Giving a different randomization seed when initializing the NMF factorization could yield an alternative set of TADs; however, we find that with NNDSVD initialization with randomized SVD, which is what GRiNCH uses, different seeds still yield a very consistent set of TADs with a median Rand Index (measuring TAD similarity from different runs with different seeds) around 0.98 (Figure S18D; Rand Index of 1 correspond to perfect agreement).*

3)	The analysis of robustness of predictions against different resolutions is important. The authors focus on the stability of predicted TAD length distribution as a measure of robustness; it would be useful to compare the predictions themselves (i.e. do the same TADs get predicted, plus or minus a fudge factor to account for varying resolution?). Also, since the number of TADs predicted is strongly determined by the choice of hyper-parameters, rather than by resolution, isn't this focus on the stability of the number of TADs predicted a bit unfair?

*Thank you for this comment and idea. In addition to comparing the length of TADs, we have now compared TADs across different resolutions by splitting lower-resolution (10kb, 25kb, and 50kb) bins into 5kb bins, the size of the lowest common denominator for different resolutions we compared. All 5kb bins “inherited” the TAD cluster assignment of their “parent” lower-resolution bin; we then measured cluster similarity on these split, higher-resolution cluster assignments (see Methods, lines 824-829). We find that GRiNCH is still one of the most stable methods under this assessment, after TopDom (see Results, lines 148-151, Figure 2C). We left in the analysis on the median TAD size and size distribution across resolutions as it still adds value in characterizing different TAD-calling methods’ behavior.*

4)	The smoothing properties of GRINCH, and its ability to detect significant interactions, should be compared against some of the many existing tools that already exist to do so.

*Thank you for this suggestion and we apologize for the confusion. Mean filter and Gaussian filter that we had initially compared GRiNCH’s smoothing properties to, have been used for pre¬processing and smoothing in various types of Hi-C data analysis, from enhancing visualization and improving the agreement among biological replicates (e.g. HiCRep from Yang et al. uses mean filter), to assisting in identifying loops (e.g., SIP from Rowley et al. uses Gaussian filter) and differential interactions (e.g., Selfish from Ardakany et al. uses Gaussian filter). We have highlighted these applications and references (line 218-221). We additionally compared GRiNCH against HiCNN, a state-of-the-art method based on convolutional neural networks, using its pretrained models for smoothing (lines 227-231 in Results, 917-930 in Methods). We find that in recovering topological structure from low-depth data, GRiNCH outperforms all other methods including HiCNN (lines 240-245, Figure 6A). In recovering significant interactions, GRiNCH outperforms unsupervised methods and HiCNN models trained on low-depth datasets (lines 254-265, Figure 6B). HiCNN’s higher performance when trained on a deeper dataset is expected as the training cell line is GM12878. We have also discussed this point in lines 426-427.*

5)	The smoothness weight lambda and dimensionality k probably has some consequences on the size distribution of predicted TADs. Is this the case? How strongly is the number of predicted TADs determined by k?

*Thank you for this question. The number of predicted TADs is in fact determined by k, as it is used both as the rank of the lower dimensional space for matrix factorization and also the number of clusters (which we interchangeably call TADs) used in the k-medoids chain-constrained clustering step (used for refining the factors and enforcing cluster contiguity, lines 536-558). To clarify this, we have updated the text in the Results, line 92-93, where we describe these hyperparameters. We demonstrated GRiNCH with three different settings for k that effectively controls the expected size of the clusters, yielding sets of clusters corresponding to subTAD- (expected size "500kb), TAD- ("1Mb), or metaTAD-scale ("2Mb) structures (lines 565¬574, Figure S14 and S15). We additionally found that different values of regularization parameters (lambda and neighborhood size) do not substantially affect the overall distribution of TAD sizes (lines 584-585, Figure S16).*

6)	How does the approach compare conceptually to Hu et al. 2016 (https://www.ncbi.nlm.nih.gov/pmc/articles/PMC4908340/ ), which is also based on non- negative matrix decomposition?

*Thank you for this question and pointer to this paper. We have cited this paper in the section introducing GRiNCH in Results (lines 76-78). There are three key conceptual differences between BNMF from Hu et al. and GRiNCH:*

*First difference lies in the high-level goals of the two approaches, which gives rise to the differences in implementation below. BNMF aims to use matrix factorization to remove the bias from the input count matrix and then use the factors directly to assign regions to clusters. BNMF does this by decomposing the input matrix into 3 factors, one of the factors is used to account for bias in the input integer interaction counts and the other two factors are used to define clusters. GRiNCH on the other hand uses matrix factorization to find contiguous region clusters and smooth a noisy matrix. GRiNCH achieves these goals by decomposing a bias-corrected input matrix into 2 factors capturing the low dimensional representation of a contact count matrix and uses the matrix completion to smooth the noisy input matrix. GRiNCH encourages smoothness of the lower dimensional representation of nearby genomic regions using graph regularization and further chain-constrained k-medoids clustering to identify contiguous genomic clusters.*

*Second, the approaches differ in the form of the objective being optimized. BNMF minimizes the KL divergence between the probability distribution of data in the input matrix and the distribution from the product of the factors. BNMF assumes the input matrix is produced by adding Poisson noise to the product of the factors. GRiNCH, on the other hand, aims to minimize the Frobenius norm of the difference between the input matrix and its approximated version (one can think of this as a version of the Euclidean distance between two matrices). The Poisson distribution, although used for count data, might be restrictive for sparse count data. GRiNCH on the other hand makes fewer assumptions about the distribution (as demonstrated from its application to Hi-C, HiChIP, SPRITE data, data of varying depths, and data from different Hi-C protocols). While both are based on NMF, BNMF’s objective assumes the matrix is symmetric. GRiNCH’s current implementation is for symmetric matrices but its objective can handle asymmetric matrices.*

*Third, due to the nature of the objective and the type of optimization BNMF needs Hu et al. note in the supplementary materials that the computational complexity of BNMF is O(kn3) with k being the lower dimension of the factors and n the size of the input matrix. The graph-regularized NMF used by GRiNCH has a lower complexity of O(kn2) – discussed further below in point 7. This difference has implications in the computing cost and resources. In a related note, Hu et al. state that the BNMF objective usually becomes stable after 1500 iterations; the GRiNCH objective usually stabilizes within 100 iterations.*

7) It would be useful to discuss the running time and memory requirement of GRINCH, especially at higher resolution.

*Thank you for this suggestion. We have added a new section “Memory consumption and runtime” (lines 588-603 in Methods) to address both the computational complexity and memory requirements of GRiNCH. Briefly, the computational complexity of graph-regularized NMF is determined by the size of the input matrix n and the lower dimension size k and is O(kn2). We also benchmarked the memory consumption (maximum resident set size) and runtime of GRiNCH across multiple combinations of input matrix size (determined by chromosome length and resolution), k (set to yield subTAD-, TAD-, or metaTAD-scale structures for each chromosome), lambda, and neighborhood radius. We find that the memory and time consumption increase in a quadratic fashion with respect to the input matrix size and in a linear fashion to k (Figure S17).*

Minor comments:

1)	What do you mean by randomly shuffle TADs?

*Thank you for calling to attention the need to clarify this procedure. Briefly, random shuffling of TADs entails taking all TADs and any non-TAD gaps between them and randomly moving them within the chromosome to preserve TAD length distribution within each method. We have provided a more detailed description of the randomization procedure in all instances they are used for generating a background/null distribution to measure significant histone enrichment (lines 761-774) and internal validation metrics values (lines 784-788, 797-805, 813-818).*

2)	"we first took the highest-depth dataset (GM12878, 86 million reads total)". This seems like a very low coverage data set? Is this only for one chromosome?

*Thank you for catching this error. These values referred to the total contact counts after post-processing steps which include filtering and square-root normalization. We have corrected the statement to “... we first took the highest-depth dataset (GM12878, 4.9 billion mapped paired-end reads) and downsampled to the depth and sparsity level of lower-depth data from other cell lines (e.g. K562, the second “deepest” cell line with 932 million reads)” in lines 156 and 158.*

*We summarize all changes to the figures in the table below:*

**Second round of review**

**Reviewer 1**

The authors have sufficiently addressed my previous comments. I think it's another useful tools for the 3D genomics community.

Plus I applaud the authors for sharing their source code prior to publication.
